# The prognosis predictive score around primary debulking surgery (PPSP) improves diagnostic efficacy in predicting the prognosis of ovarian cancer

**DOI:** 10.1038/s41598-022-27333-1

**Published:** 2022-12-31

**Authors:** Naoki Kawahara, Ryuji Kawaguchi, Keita Waki, Tomoka Maehana, Shoichiro Yamanaka, Yuki Yamada, Fuminori Kimura

**Affiliations:** Department of Obstetrics and Gynecology, Nara Medical University, 840 Shijo-cho, Kashihara, 634-8522 Japan

**Keywords:** Ovarian cancer, Tumour biomarkers

## Abstract

In recent years, the pretreatment inflammatory responses have proven to predict the prognosis, but no report exists analyzing the combined inflammatory response of the pre- and postsurgical treatment. The current study aims to extract the factors predicting the recurrence and create novel predictive scoring. This retrospective study was conducted at our institution between November 2006 and December 2020, with follow-up until September 2022. Demographic and clinicopathological data were collected from women who underwent primary debulking surgery. We created the scoring system named the prognosis predictive score around primary debulking surgery(PPSP) for progression-free survival(PFS). Univariate and multivariate analyses were performed to assess its efficacy in predicting PFS and overall survival(OS). Cox regression analyses were used to assess its time-dependent efficacy. Kaplan–Meier and the log-rank test were used to compare the survival rate. A total of 235 patients were included in the current study. The cut-off value of the scoring system was six. Multivariate analyses revealed that an advanced International Federation of Gynecology and Obstetrics(FIGO) stage (p < 0.001 for PFS; p = 0.038 for OS), the decreased white blood cell count difference (p = 0.026 for PFS) and the high-PPSP (p = 0.004 for PFS; p = 0.002 for OS) were the independent prognostic factors. Cox regression analysis also supported the above results. The PPSP showed good prognostic efficacy not only in predicting the PFS but also OS of ovarian cancer patients comparable to FIGO staging.

## Introduction

Ovarian cancer is women's fifth leading cause of cancer-related death^1^. Because patients have relatively few symptoms in the early stages and most ovarian cancer cases are diagnosed at advanced stages, this disease is called the silent killer^2–7^. Over 185,000 deaths from this disease are reported annually worldwide^8,9^. Ovarian cancer is divided into epithelial, germ cell, and sex cord-stromal tumors, and epithelial ovarian cancer, which have the highest rate at over 90%^10,11^. The age of onset is mainly in the post-menopause^12,13^, and the overall survival rate according to the International Federation of Gynecology and Obstetrics(FIGO) stage for I, II, and III/IV were reported as 74.5%, 54.5%, and 24.7% respectively^14^. The recurrence rate rises according to the FIGO stage and advanced stages as III and IV show a high recurrence rate of approximately 80%^15^. Ovarian cancer is strongly recommended to resect the tumor as possible because the residual tumor is related to lower progression-free survival(PFS) and overall survival(OS)^16,17^. Thus operable ovarian cancer is treated with surgical resection in advance (i.e., PDS: Primary Debulking Surgery[PDS]) followed by postoperative adjuvant chemotherapy^18,19^.

In recent years, inflammatory reactions in the tumor microenvironment have been shown to play an important role in tumor development and progression^20,21^. Peripheral leukocytes, neutrophils, lymphocytes, platelets, and acute-phase proteins contribute to the inflammatory response and can be detected easily. A number of studies have demonstrated that the systemic inflammatory response is related to the overall survival of surgically treated cancer patients^22–24^. Some pre-treatment indexes, such as the tumor-related leukocytosis(TRL)^25,26^, neutrophil/lymphocyte ratio(NLR)^27–29^, platelet/lymphocyte ratio(PLR)^27,30,31^, monocyte/lymphocyte ratio(MLR)^32,33^, Glasgow prognostic score/modified Glasgow prognostic score(GPS/mGPS)^34–36^, and systemic immune-inflammation index (SII)^37,38^ have been shown to have good prognostic value. In this context, it is suggested that the tumor microenvironment has an extraordinary effect on the systemic immune system, and reduced inflammatory status after surgery should be a strong impact on the prognosis. However, no predictive scoring system exists based on pre- and post-PDS predictive factors. Actually, patients underwent surgery have to wait nervously for the effect of the adjuvant chemotherapy and the physician has to follow up strictly with all patients. This study aims to seek the prognostic factors related to recurrence around PDS in ovarian cancer, create the prognostic score predicting the prognosis of post-PDS ovarian cancer, and analyze the usefulness of the scoring.

## Results

**Figure 1:**
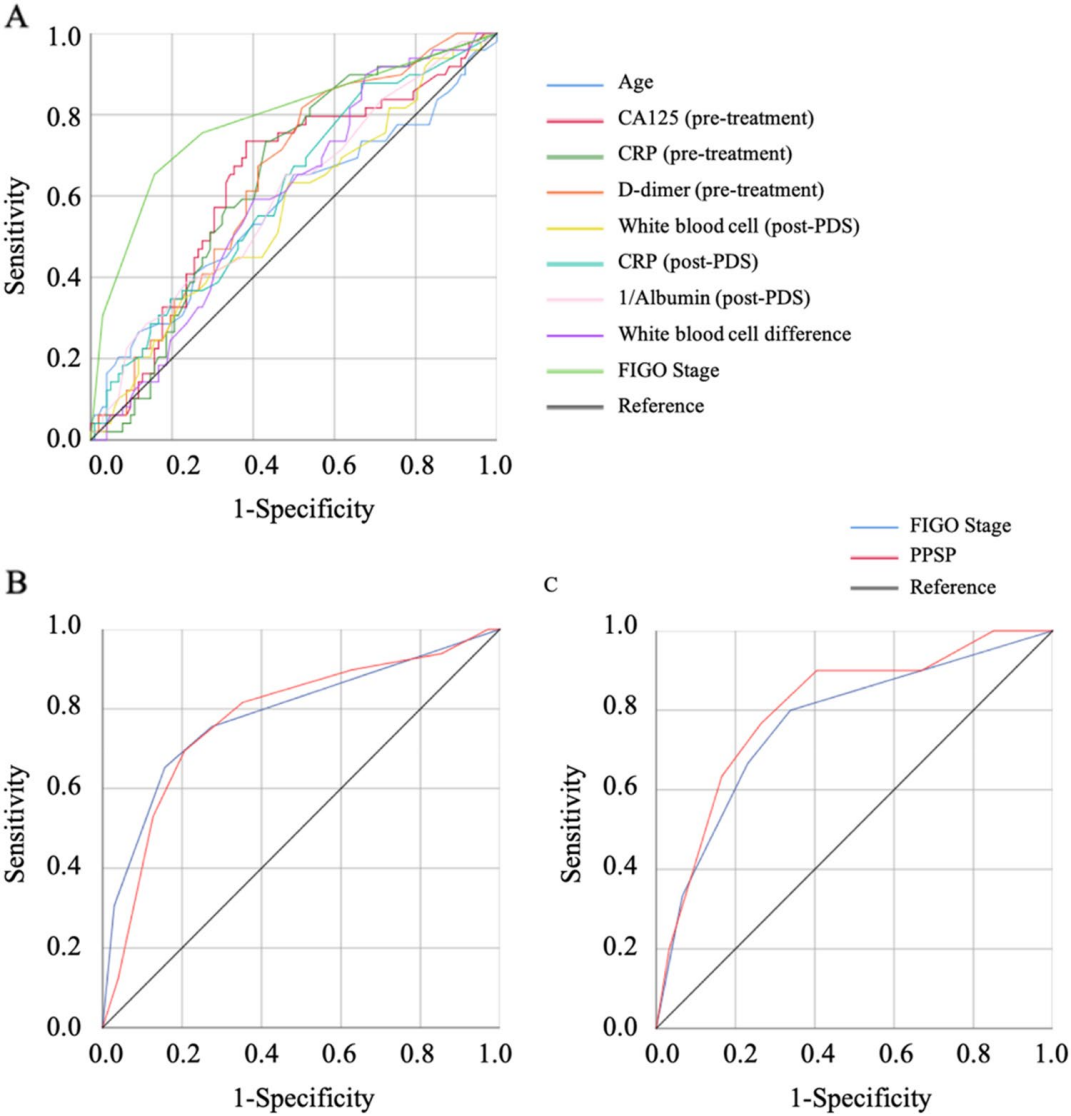
The ROC curves of each factors in the current cohort. All factors showed a high AUC with significant differentiation (**A**). The FIGO staging marked highest AUC. The PPSP showed slightly lower AUC than the FIGO staging for PFS (0.776 vs. 0.809) (**B**), and the PPSP and the FIGO staging showed similar AUC value for OS (0.809 vs. 0.806) (**C**).

**Figure 2:**
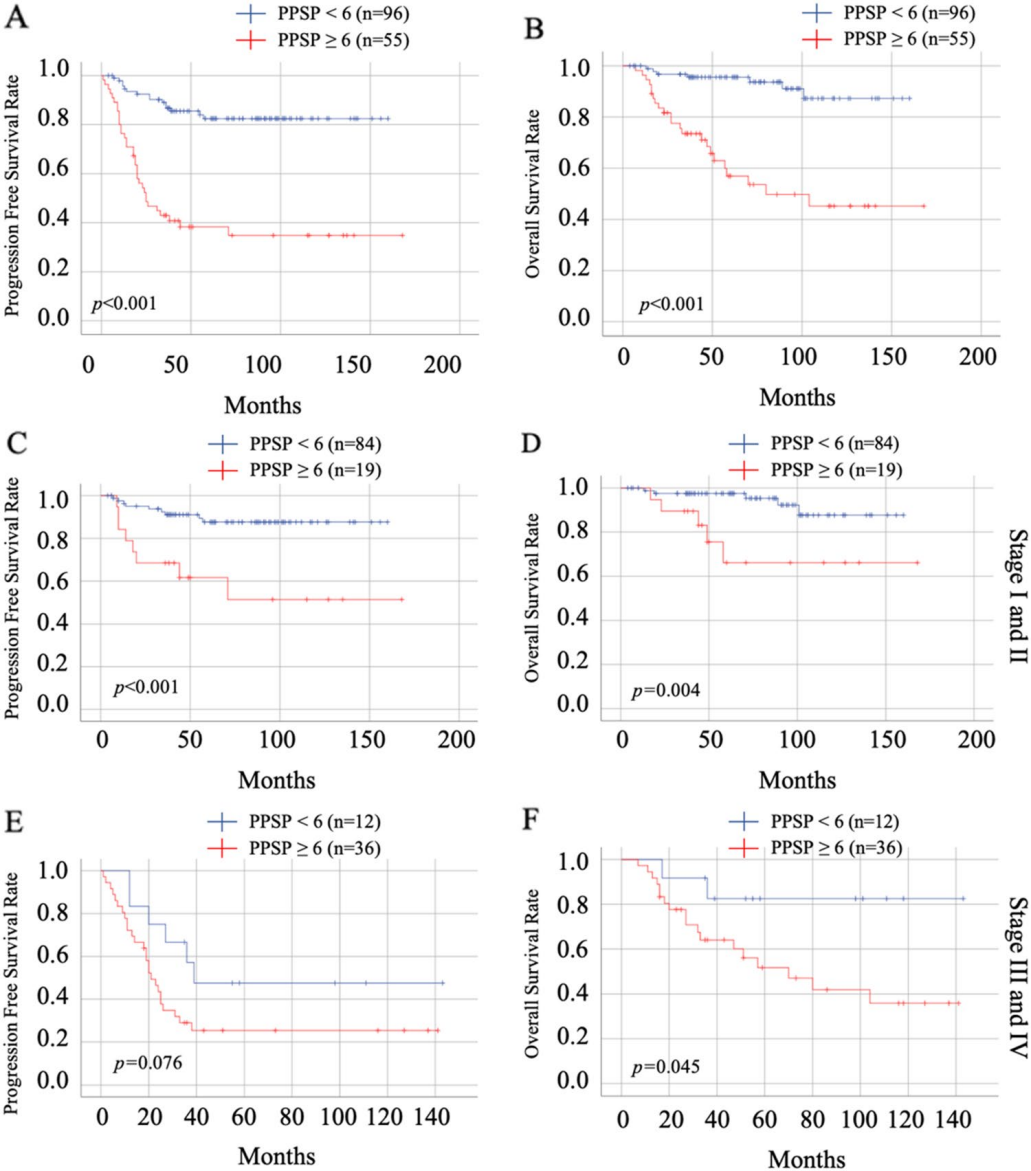
Log lank analysis revealed low-PPSP (< 6) showed good prognostic efficacy in both PFS (**A**) and OS (**B**) (p < 0.001). Divided into I/II or III/IV according to FIGO staging, PPSP showed good efficacy to predict PFS (p < 0.001) in I/II stages (**C**) and OS in both groups (p = 0.004 and p = 0.045) (**D,F**). However it did not reach significant differentiation to predict PFS in III/IV stages (**E**).

From November 2006 and December 2020, a total of 235 patients were included in this study. Patient's peripheral blood data were collected at the first hospitalization before and after PDS, and the median days from PDS were 25 days. A total of 183(77.8%) patients underwent chemotherapy after surgery. Among the patients who did not underwent chemotherapy, 45(86.5%) patients were the stage I. The recurrence and non-recurrence cases were 68(28.9%) and 167(71.1%) cases, respectively. The demographic and clinical characteristics of the current cohort are outlined in Table 1. The recurrence cases showed trends in older age and advanced stages. Serous-type tumor tended to have higher recurrence rate than other tumor subtypes. In the current cohort, there was no significant differentiation in the distribution of peripheral blood cells before PDS (Table2). The carbohydrate antigen125(CA125), C-reactive protein(CRP), and the D-dimer reached significant differentiation between the non-recurrent and recurrent patients. The results of the ROC curve analysis bases on the detection of recurrence are shown in Table 3. The optimal cutoff value was determined by analyzing the ROC curve predicting the recurrence. The ROC analysis showed the same result as peripheral blood markers before treatment, white blood cell counts, CRP, and albumin after PDS showed an efficacy. Moreover, the difference in white blood cell counts showed efficacy (Table 3, Fig. 1A). Table 4 shows the distribution of the above candidates related to pre- and post-PDS assessment. PPSP is defined by older age (≥ 55 years), elevated pretreatment CA125 (≥ 124.5 U/mL), pretreatment CRP (≥ 0.26 mg/dL), and pretreatment D-dimer (≥ 1.1 µg/mL), and post-PDS white blood cell count (≥ 57.00 × 10^2^/µL), post-PDS CRP (≥ 0.08 mg/dL), post-PDS hypo-albuminemia (< 4.0 g/dL), and white blood cell counts difference ([post-PDS counts – pre-pretreatment counts] ≥ –29.00 × 10^2^/µL), if all parameters are abnormal, the assigned value is 8; and if all parameters are normal, the assigned value is 0. We next assessed the efficacy of the PPSP in discriminating between non-recurrent and recurrent cases. The result of the ROC curve analysis based on the discriminating non-recurrent and recurrent cases is shown in Fig. 1B,C. The cut-off value from the above scoring was six for PFS and OS (sensitivity: 69.4%, specificity: 79.4%, AUC = 0.776, p < 0.001; sensitivity: 76.7%, specificity: 73.6%, AUC = 0.804, p < 0.001, respectively) (Fig. 1B,C). A multivariate analysis confirmed that the FIGO stage, white blood cell difference, and the PPSP were extracted as independent factors for predicting recurrence (Risk ratio[RR]: 5.48, 95% confidence interval(CI): 2.14–14.02, p < 0.001; RR: 4.04, 95% CI: 1.18–13.86, p = 0.026; RR: 3.85, 95% CI: 1.54–9.65, p = 0.004, respectively)(Table 5). For predicting mortality, FIGO stage and the PPSP were extracted as independent factors (RR: 2.91, 95% CI: 1.06–7.96, p = 0.038; RR: 5.71, 95% CI: 1.86–15.50, p = 0.002, respectively)(Table 6). Cox regression analyses revealed that an advanced FIGO stage (Hazard ratio[HR]: 3.27, 95% CI: 1.60–6.67, p = 0.001 for PFS; HR: 2.45, 95% CI: 1.03–5.82, p = 0.042 for OS), white blood cell difference (HR: 3.30, 95% CI: 1.17–9.23, p = 0.023 for PFS), and high-PPSP (HR: 2.99, 95% CI: 1.43–6.23, p = 0.003 for PFS; HR: 4.55, 95% CI: 1.73–11.97, p = 0.002 for OS) were the independent prognostic factors. Log lank analysis revealed that low-PPSP (< 6) showed good prognostic efficacy in both PFS and OS (p < 0.001)(Fig. 2A,B). Even divided into early or advanced stages according to FIGO staging as I/II or III/IV, PPSP showed good efficacy to predict PFS and OS other than PFS in stage III/IV (Fig. 2C–F).

**Table 1:** Demographic and clinical characteristics of the current cohort.

	Non-recurrence	Recurrence	*p*-value
Number	*n* = 167	*n* = 68	
**Age (years)**			
Median (range)	55.00 (17–88)	60.50 (35–86)	
Mean ± SD	55.91 ± 12.82	60.26 ± 12.60	0.029
**BMI**			
Median (range)	21.57 (15.37–40.80)	22.00 (16.60–35.15)	
Mean ± SD	22.41 ± 4.13	22.44 ± 4.28	0.908
**Parity**			
0	61	14	
≥ 1	104	52	0.021
**FIGO stage**			
I	122	13	
II	18	9	
III	23	28	
IV	4	18	< 0.001
**TMN classification**			
pT1	1a (45), 1b (2), 1c (81)	1a (2), 1b (0), 1c (19)	
pT2	2a (4), 2b (17)	2a (4), 2b (9)	
pT3	3a (0), 3b (7), 3c (11)	3a (1), 3b (8), 3c (25)	< 0.001
pN	0 (101), 1 (10)	0 (24), 1 (17)	< 0.001
pM	0 (162), 1 (4)	0 (50), 1 (18)	< 0.001
**Tumor subtype**			
Serous	29	28	
Endometrioid	45	6	
Clear cell	53	13	
Mucinous	16	8	
Seromucinous	4	1	
Mixed	3	2	
Others	17	10	0.001

*BMI* body mass index, *FIGO* The International Federation of Gynecology and Obstetrics.

**Table 2:** Pre-treatment peripheral blood cell distributions.

	Non-recurrence	Recurrence	*p*-value
Number	*n* = 167	*n* = 68	
**Hemoglobin (g/mL)**			
Median (range)	12.70 (4.6–16.3)	12.20 (8.0–14.3)	
Mean ± SD	12.40 ± 1.76	12.10 ± 1.48	0.110
**Platelet (× 10**^**4**^**/µL)**			
Median (range)	27.30 (9.2–64.0)	28.35 (13.8–70.0)	
Mean ± SD	28.28 ± 8.73	30.96 ± 11.01	0.169
**White blood cell (× 10**^**2**^**/µL)**			
Median (range)	67.00 (25.00–2.09 × 10^2^)	66.50 (35.00–1.67 × 10^2^)	
Mean ± SD	74.94 ± 32.27	69.77 ± 22.48	0.679
**Neutrophils (%)**			
Median (range)	69.00 (41.1–94.1)	70.30 (28.9–88.6)	
Mean ± SD	68.48 ± 12.01	69.60 ± 10.51	0.435
**Neutrophils (× 10**^**2**^**/µL)**			
Median (range)	46.13 (16.02–1.89 × 10^2^)	45.27 (13.58–1.41 × 10^2^)	
Mean ± SD	54.59 ± 32.33	50.13 ± 22.18	0.982
**Lymphocytes (%)**			
Median (range)	19.80 (2.3–46.2)	20.30 (4.5–43.0)	
Mean ± SD	21.62 ± 10.20	20.74 ± 8.24	0.781
**Lymphocytes (× 10**^**2**^**/µL)**			
Median (range)	13.68 (3.07–32.62)	13.27 (4.63–33.54)	
Mean ± SD	14.58 ± 6.12	13.49 ± 4.79	0.348
**Monocytes (%)**			
Median (range)	6.20 (1.2–12.6)	6.10 (2.5–13.2)	
Mean ± SD	6.22 ± 2.12	6.28 ± 2.21	0.943
**Monocytes (× 10**^**2**^**/µL)**			
Median (range)	4.04 (1.11–11.60)	4.15 (1.22–8.84)	
Mean ± SD	4.41 ± 1.86	4.24 ± 1.56	0.882
**CA125 (U/mL)**			
Median (range)	79.00 (7–15.45 × 10^3^)	2.11 × 10^2^ (8–18.33 × 10^3^)	
Mean ± SD	6.88 × 10^2^ ± 19.22 × 10^2^	13.84 × 10^2^ ± 34.65 × 10^2^	0.005
**CEA (ng/mL)**			
Median (range)	2.50 (0.3–1.88 × 10^2^)	1.90 (0.3–98.9)	
Mean ± SD	6.88 ± 19.20	6.44 ± 16.59	0.345
**CA 19-9 (U/mL)**			
Median (range)	17.50 (1.0–93.92 × 10^3^)	15.00 (1.0–15.26 × 10^2^)	
Mean ± SD	10.88 × 10^2^ ± 78.49 × 10^2^	92.68 ± 2.30 × 10^2^	0.292
**CRP (mg/dL)**			
Median (range)	0.20 (0.00–26.00)	0.75 (0.00–27.28)	
Mean ± SD	1.76 ± 3.61	2.04 ± 4.11	0.004
**Albumin (g/dL)**			
Median (range)	4.20 (1.3–7.3)	4.10 (2.7–5.0)	
Mean ± SD	4.20 ± 0.55	4.11 ± 0.49	0.128
**d-dimer (µg/mL)**			
Median (range)	1.20 (0.4–56.6)	2.20 (0.5–34.7)	
Mean ± SD	4.11 ± 7.44	4.81 ± 6.50	0.007

*Hb* hemoglobin, *CA125* carbohydrate antigen125, *CEA* carcinoembryonic antigen, *CA 19-9* carbohydrate antigen 19-9, *CRP* C-reactive protein.

**Table 3:** The cut-off values predicting recurrence.

	AUC	*p*-value	Cut-off value	Sensitivity	Specificity	PPV	NPV
Age	0.591	0.029	55	0.676	0.491	35.11	78.85
CA125 (pre-treatment)	0.672	0.005	124.5	0.672	0.618	41.67	82.26
CRP (pre-treatment)	0.619	0.004	0.26	0.691	0.575	39.83	82.05
d-dimer (pre-treatment)	0.619	0.007	1.1	0.803	0.467	40.50	84.00
White blood cell (post-PDS)	0.592	0.028	5700	0.582	0.588	36.44	77.60
CRP (post-PDS)	0.603	0.016	0.08	0.875	0.342	34.56	87.30
Albumin (post-PDS)	0.621	0.010	4.0	0.655	0.532	38.29	77.64
White blood cell difference	0.594	0.025	–2900	0.896	0.321	34.26	88.88

*CA125* carbohydrate antigen125, *CRP* C-reactive protein, *Hb* hemoglobin, *PDS *primary debulking surgery, *PPV* positive predictive value, *NPV* negative predictive value, *AUC* area under curve.

**Table 4:** Post-PDS peripheral blood cell and serum markers.

	Non-recurrence	Recurrence	*p*-value
Number	*n* = 167	*n* = 68	
**White blood cell (post-PDS) (× 10**^**2**^**/µL)**			
Median (range)	54.00 (18.00–1.14 × 10^2^)	58.00 (27.00–2.29 × 10^2^)	
Mean ± SD	54.31 ± 15.53	62.02 ± 28.07	0.028
**CRP (post-PDS) (mg/dL)**			
Median (range)	0.13 (0.00–14.26)	0.30 (0.00–11.20)	
Mean ± SD	0.67 ± 1.62	1.14 ± 2.12	0.015
**Albumin (post-PDS) (g/dL)**			
Median (range)	4.00 (2.6–4.9)	3.80 (2.2–4.8)	
Mean ± SD	3.93 ± 0.49	3.70 ± 0.54	0.010
**White blood cell difference (× 10**^**2**^**/µL)**			
Median (range)	–13.00 (–1.40 × 10^2^–62.00)	–7.00 (–74.00–1.62 × 10^2^)	
Mean ± SD	–20.67 ± 31.74	–7.77 ± 28.13	0.001

*CRP* C-reactive protein, *PDS* primary debulking surgery.

**Table 5:** Univariate and multivariable analysis of the predictive factors of recurrence.

		Univariate analysis		Multivariate analysis	
		Risk ratio (95% CI)	*p*-value	Risk ratio (95% CI)	*p*-value
Age (years)	< 55	1.00 (referent)			
	≥ 55	2.01 (1.11–3.64)	0.020		
FIGO stage	< 3	1.00 (referent)		1.00 (referent)	
	≥ 3	10.84 (5.63–20.85)	< 0.001	5.48 (2.14–14.02)	< 0.001
CA125 (pre-treatment) (U/mL)	< 124.5	1.00 (referent)			
	≥ 124.5	3.31 (1.82–6.02)	< 0.001		
CRP (pre-treatment) (mg/dL)	< 0.26	1.00 (referent)			
	≥ 0.26	3.02 (1.66–5.50)	< 0.001		
d-dimer (pre-treatment) (µg/mL)	< 1.1	1.00 (referent)			
	≥ 1.1	3.57 (1.74–7.31)	< 0.001		
White blood cell	< 57.00	1.00 (referent)			
(post-PDS) (× 10^2^/µL)	≥ 57.00	1.98 (1.11–3.53)	0.019		
CRP (post-PDS)	< 0.08	1.00 (referent)			
(mg/dL)	≥ 0.08	3.63 (1.61–8.15)	0.002		
Albumin (post-PDS)	≥ 4.0	1.00 (referent)			
(g/dL)	< 4.0	2.15 (1.11–4.16)	0.022		
White blood cell difference (× 10^2^/µL)	< –29.00	1.00 (referent)		1.00 (referent)	
	≥ –29.00	4.17 (1.69–10.29)	0.002	4.04 (1.18–13.86)	0.026
PPSP	< 6	1.00 (referent)		1.00 (referent)	
	≥ 6	8.74 (4.03–18.96)	< 0.001	3.85 (1.54–9.65)	0.004

*FIGO* The International Federation of Gynecology and Obstetrics, *CA125* carbohydrate antigen125, *CRP* C-reactive protein, *PDS* primary debulking surgery, *PPSP* prognosis predictive score around PDS.

**Table 6:** Univariate and multivariable analysis of the predictive factors of mortality.

		Univariate analysis		Multivariate analysis	
		Risk ratio (95% CI)	*p*-value	Risk ratio (95% CI)	*p*-value
Age (years)	< 55	1.00 (referent)			
	≥ 55	1.83 (0.93–3.62)	0.079		
FIGO stage	< 3	1.00 (referent)		1.00 (referent)	
	≥ 3	8.25 (4.02–16.90)	< 0.001	2.91 (1.06–7.96)	0.038
CA125 (pre-treatment) (U/mL)	< 124.5	1.00 (referent)			
	≥ 124.5	3.43 (1.69–6.99)	0.001		
CRP (pre-treatment) (mg/dL)	< 0.26	1.00 (referent)			
	≥ 0.26	3.54 (1.72–7.26)	0.001		
d-dimer (pre-treatment) (µg/mL)	< 1.1	1.00 (referent)			
	≥ 1.1	5.69 (2.12–15.31)	0.001		
White blood cell (post-PDS) (× 10^2^/µL)	< 57.00	1.00 (referent)			
	≥ 57.00	3.23 (1.61–6.48)	0.001		
CRP (post-PDS) (mg/dL)	< 0.08	1.00 (referent)			
	≥ 0.08	3.43 (1.28–9.18)	0.014		
Albumin (post-PDS) (g/dL)	≥ 4.0	1.00 (referent)			
	< 4.0	2.86 (1.28–6.38)	0.010		
White blood cell difference (× 10^2^/µL)	< –29.00	1.00 (referent)			
	≥ –29.00	2.84 (1.06–7.60)	0.038		
PPSP	< 6	1.00 (referent)		1.00 (referent)	
	≥ 6	9.13 (3.57–23.33)	< 0.001	5.71 (1.86–15.50)	0.002

*FIGO* The International Federation of Gynecology and Obstetrics, *CA125* carbohydrate antigen125, *CRP* C-reactive protein, *PDS* primary debulking surgery, *PPSP* prognosis predictive score around PDS.

## Discussion

Several studies have been reported to predict PFS and OS in ovarian cancer using pre-treatment factors, at least to our knowledge there is no prognostic scoring system consisting both of pre- and post-PDS patients’ data. The current study revealed that the PPSP showed great efficacy in predicting PFS and OS, which were comparable to FIGO staging.

The CA125 was considered the most promising serum marker of ovarian cancer^39^. It has been thought that higher preoperative serum CA125 levels are directly related to a larger tumor burden^40,41^, and there have been numerous discussions about whether the CA125 level could predict optimal surgical cytoreduction^42^. In this context, CA125 reflects not only the tumor burden but also the carcinomatosis^43–45^. In the current study, the pre-treatment CA125 was extracted in the scoring system regardless of tumor subtype, which could reflect the peritoneal inflammation rather than tumor burden, partly because the current study did not include only CA125 productive tumors. CRP is synthesized by hepatocytes. It is a non-specific yet sensitive marker of acute inflammatory response and is expressed in selected neoplastic cells^46^. Numerous studies have indicated that an increased CRP level value indicates poor prognosis in various types of cancer^47–50^. Albumin, similarly, is generally used for assessing nutritional status^46^. Malnutrition and inflammation suppress albumin synthesis, thereby reducing immune defense, impeding treatment response, and contributing to adverse outcomes in patients with cancer^51^. Malignant tumors also consume such nutrition as albumin^52^, leading to edema and cachexia, which have been reported to be correlated with an unfavorable prognosis for some gastrointestinal tumors^53,54^. Moreover, the GPS, a cumulative inflammation-based cancer-prognostic marker composed of serum elevation of CRP and decrease in albumin concentration, is likely to reflect host systemic inflammatory response and has been reported to be significant as a prognostic indicator in cancer-bearing patients^55–57^. In the current study, these CRP and albumin were also extracted as a candidate for prognosis poor outcomes in ovarian cancer patients, comparable to these reports. d-dimer, a soluble fibrin-degradation product, is a valuable marker for diagnosing venous thromboembolism^58^. The d-dimer test is frequently positive for venous thromboembolism and inflammatory autoimmune disease as rheumatoid arthritis, cancer, elderly age, surgery, trauma, pregnancy, and postpartum. We previously reported that a high pre-treatment plasma d-dimer level was one of the independent risk factors of overall survival^59^. d-dimer could be another significant inflammatory factor that predicts the outcome of ovarian cancer.

Numerous reports, on ovarian cancer, have created evidence that NLR, LMR, and PLR including platelet count may be helpful indicators for differentiating benign neoplasms from malignant changes^60,61^. Moreover, they are sensitive indicators correlated with local advancement and response to first-line chemotherapy. However, we did not find the effectiveness of the true platelet, neutrophil, monocyte, and lymphocyte counts. Instead, we found the prognostic evidence of post-PDS white blood cell counts and their difference. This scoring system shared rather the factors with GPS/mGPS^34,36^ and leukocytosis^25,26^ than NLR, LMR, and PLR^60^. This method could be more useful for the physician.

This study has some limitations. The first limitation is that we did not compare the PPSP with such predictive scoring as NLR, LMR, PLR, GPS/mGPS, and SII as a nature of new reporting of the novel scoring system. Second, we did not investigate the cases of interval debulking surgery cases mainly administrated in firstly inoperative cases because the peripheral blood counts were dramatically altered by the chemotherapy. We will report a novel scoring system around interval debulking surgery in the near future.

In conclusion, The PPSP showed good prognostic efficacy not only in predicting the PFS but also OS of ovarian cancer patients comparable to FIGO staging.

## Methods

### Patients

A list of patients with primary, previously untreated, histologically-confirmed ovarian cancers who were treated at Nara Medical University Hospital between November 2006 and December 2020 was generated from our institutional registry. They were followed-up until September 2022. The study was conducted according to the guidelines of the Declaration of Helsinki, and approved by the Institutional Ethics Committee of Nara Medical University Hospital (protocol code: 3377).We included in this study the cases who underwent PDS. All cases were histologically confirmed. Written informed consent to use the patient's clinical data for research was obtained at the first hospitalization, and after approval by the Ethics Review Committee of the Nara Medical Hospital, the opt-out form was provided through our institutional homepage. A total of 235 patients were included in the current cohort. No patients had undergone chemotherapy or radiotherapy for ovarian tumors before treatment. The following factors were collected through a chart review of the patient's medical records: age, body mass index(BMI), parity, postoperative diagnosis including FIGO stage, TNM classifications, tumor subtypes, and pre-treatment and post-PDS blood test results. Post-PDS blood test was conducted on the first outpatient visit after PDS. Factors after PDS were analyzed either the values themselves or the difference which is calculated by subtraction pretreatment value from the PDS.

### Statistical analysis

Analyses were performed using SPSS version 25.0 (IBM SPSS, Armonk, NY, USA). The differences of each factor were compared using a Mann–Whitney U test. The receiver operating characteristic(ROC) curve analysis was performed to determine the cut-off value for predicting poor prognosis. The cut-off value was based on the highest Youden index (i.e., sensitivity + specificity − 1). We used a logistic regression analysis to assess the risk factors for poor prognosis. And to assess its time dependent prognosis efficacy cox regression analyses and log rank test were selected. A two-sided p < 0.05 was considered as indicating a statistically significant difference.

## Data Availability

The datasets generated during and analyzed during the current study are available from the corresponding author on reasonable request.

## References

[1] Siegel RL, Miller KD, Jemal A (2019). Cancer statistics, 2019. CA Cancer J. Clin..

[2] Bharwani N, Reznek RH, Rockall AG (2011). Ovarian Cancer Management: The role of imaging and diagnostic challenges. Eur. J. Radiol..

[3] Saorin A (2020). Emerging role of metabolomics in ovarian cancer diagnosis. Metabolites.

[4] Feeney L (2020). Liquid biopsy in ovarian cancer: Catching the silent killer before it strikes. World J. Clin. Oncol..

[5] Zhang Z (2004). Three biomarkers identified from serum proteomic analysis for the detection of early stage ovarian cancer. Cancer Res..

[6] Stewart C, Ralyea C, Lockwood S (2019). Ovarian cancer: An integrated review. Semin. Oncol. Nurs..

[7] Lheureux S (2019). Epithelial ovarian cancer. Lancet.

[8] Perrone MG (2020). Translational theragnosis of ovarian cancer: Where do we stand?. Curr. Med. Chem..

[9] Zampieri LX (2020). Mitochondria participate in chemoresistance to cisplatin in human ovarian cancer cells. Mol. Cancer Res..

[10] Torre LA (2018). Ovarian cancer statistics, 2018. CA Cancer J Clin..

[11] Debuquoy C (2020). Rare ovarian tumors: An update on diagnosis and treatment. Int J Gynecol Cancer..

[12] Jayson GC (2014). Ovarian cancer. Lancet.

[13] Davenport CF (2022). Diagnostic models combining clinical information, ultrasound and biochemical markers for ovarian cancer: Cochrane systematic review and meta-analysis. Cancers.

[14] Dinkelspiel HE (2015). Long-term mortality among women with epithelial ovarian cancer. Gynecol. Oncol..

[15] Rose PG (2022). Ovarian cancer recurrence: Is the definition of platinum sensitivity modified by PARPi, bevacizumab or other intervening treatments? : A clinical perspective. Cancer Drug Resist..

[16] Orr B, Edwards RP (2018). Diagnosis and treatment of ovarian cancer. Hematol. Oncol. Clin. N. Am..

[17] Narod S (2016). Can advanced-stage ovarian cancer be cured?. Nat. Rev. Clin. Oncol..

[18] Eisenhauer EA (2017). Real-world evidence in the treatment of ovarian cancer. Ann. Oncol..

[19] Lawrie TA (2015). Adjuvant (post-surgery) chemotherapy for early stage epithelial ovarian cancer. Cochrane Database Syst. Rev..

[20] Ostan R (2015). Inflammaging and cancer: A challenge for the Mediterranean diet. Nutrients.

[21] Candido J, Hagemann T (2013). Cancer-related inflammation. J. Clin. Immunol..

[22] Lin JX (2021). Prognostic importance of dynamic changes in systemic inflammatory markers for patients with gastric cancer. J. Surg. Oncol..

[23] Holub K (2020). Analysis of systemic inflammatory factors and survival outcomes in endometrial cancer patients staged I–III FIGO and treated with postoperative external radiotherapy. J. Clin. Med..

[24] Dolan RD (2017). The role of the systemic inflammatory response in predicting out-comes in patients with operable cancer: Systematic review and meta-analysis. Sci. Rep..

[25] So KA (2014). The prognostic significance of preoperative leukocytosis in epithelial ovarian carcinoma: A retrospective cohort study. Gynecol. Oncol..

[26] Barber EL (2015). Association of preoperative thrombocytosis and leukocytosis with postoperative morbidity and mortality among patients with ovarian cancer. Obstet. Gynecol..

[27] Leng J, Wu F, Zhang L (2022). Prognostic significance of pretreatment neutrophil-to-lymphocyte ratio, platelet-to-lymphocyte ratio, or monocyte-to-lymphocyte ratio in endometrial neoplasms: A systematic review and meta-analysis. Front. Oncol..

[28] Chen G (2018). Prognostic role of neutrophil to lymphocyte ratio in ovarian cancer: A meta-analysis. Technol. Cancer Res. Treat..

[29] Huang QT (2017). Prognostic significance of neutrophil-to-lymphocyte ratio in ovarian cancer: A systematic review and meta-analysis of observational studies. Cell Physiol. Biochem..

[30] Tian C (2018). Prognostic significance of platelet-to-lymphocyte ratio in patients with ovarian cancer: A me-ta-analysis. Eur. J. Clin. Invest..

[31] Ma XM (2017). The platelet-to-lymphocyte ratio as a predictor of patient outcomes in ovarian cancer: A meta-analysis. Climacteric.

[32] Kwon BS (2018). Prognostic value of preoperative lymphocyte–monocyte ratio in patients with ovarian clear cell carcinoma. J. Cancer..

[33] Eo WK (2016). The lymphocyte–monocyte ratio predicts patient survival and aggressiveness of ovarian cancer. J. Cancer.

[34] Zhu J (2016). The Glasgow Prognostic Score (GPS) is a novel prognostic indicator in advanced epithelial ovarian cancer: A multicenter retrospective study. J. Cancer Res. Clin. Oncol..

[35] Roncolato FT (2018). Validation of the modified Glasgow Prognostic Score (mGPS) in recurrent ovarian cancer (ROC)—Analysis of patients enrolled in the GCIG symptom benefit study (SBS). Gynecol. Oncol..

[36] Omichi C (2016). Glasgow prognostic score is an independent marker for poor prognosis with all cases of epithelial ovarian cancer. Cancer Med..

[37] Nie D (2019). Systemic immune-inflammation index predicts prognosis in patients with epithelial ovarian cancer: A retrospective study. Gynecol. Oncol..

[38] Ramón-Rodríguez J (2022). Prognostic value of pre-operative systemic immune-inflammation index and platelet to lymphocyte ratio in peritoneal carcinomatosis of ovarian origin. Surg. Oncol..

[39] Ahmed AA, Abdou AM (2019). Diagnostic accuracy of CA125 and HE4 in ovarian carcinoma patients and the effect of confounders on their serum levels. Curr. Probl. Cancer.

[40] Maughan TS (1988). Antigen CA125 in tumor tissue and serum from patients with adenocarcinoma of the ovary. Gynecol. Oncol..

[41] Ayhan A (2007). Is there a correlation between tumor marker panel and tumor size and histopathology in well staged patients with borderline ovarian tumors?. Acta Obstet. Gynecol. Scand..

[42] Memarzadeh S (2003). CA125 levels are a weak predictor of optimal cytoreductive surgery in patients with advanced epithelial ovarian cancer. Int. J. Gynecol. Cancer.

[43] Duzgun O, Sarici IS (2019). Preoperative CA125 value predicts Glisson capsule involvement in patients with peritoneal carcinomatosis undergoing cytoreductive surgery and hyperthermic intraperitoneal chemotherapy. Biomark. Med..

[44] Saygili U (2002). The effect of ascites, mass volume, and peritoneal carcinomatosis on serum CA125 levels in patients with ovarian carcinoma. Int. J. Gynecol. Cancer.

[45] Diaz-Gil D (2016). Prediction of 5-year survival in advanced-stage ovarian cancer patients based on computed tomography peritoneal carcinomatosis index. Abdom. Radiol. (NY).

[46] Gabay C, Kushner I (1999). Acute-phase proteins and other systemic responses to inflammation. N. Engl. J. Med..

[47] Jones JM (2006). Plasma fibrinogen and serum C-reactive protein are associated with non-small cell lung cancer. Lung Cancer.

[48] Hashimoto K (2005). The impact of preoperative serum C-reactive protein on the prognosis of patients with hepatocellular carcinoma. Cancer.

[49] Crumley ABC (2006). An elevated C-reactive protein concentration, prior to surgery, predicts poor cancer-specific survival in patients undergoing resection for gastro-oesophageal cancer. Br. J. Cancer.

[50] Hefler LA (2008). Serum C-reactive protein as independent prognostic variable in patients with ovarian cancer. Clin. Cancer Res..

[51] Mantzorou M (2017). Clinical value of nutritional status in cancer: What is its impact and how it affects disease progression and prognosis?. Nutr. Cancer.

[52] Galenkamp KMO, Alas B, Commisso C (2019). Quantitation of macropinocytosis in cancer cells. Methods Mol. Biol..

[53] Liu XY (2021). One-year mortality in patients with cancer cachexia: Association with albumin and total protein. Cancer Manag. Res..

[54] Bachmann J (2008). Cachexia worsens prognosis in patients with resectable pancreatic cancer. J. Gastrointest. Surg..

[55] Proctor MJ (2011). An inflammation-based prognostic score (mGPS) predicts cancer survival independent of tumour site: A Glasgow Inflammation Outcome Study. Br. J. Cancer.

[56] Read JA (2006). Evaluation of nutritional and inflammatory status of advanced colorectal cancer pa-tients and its correlation with survival. Nutr. Cancer..

[57] Nozoe T (2011). Significance of modified Glasgow prognostic score as a useful indicator for prognosis of patients with gastric carcinoma. Am. J. Surg..

[58] Weitz JI, Fredenburgh JC, Eikelboom JW (2017). A test in context: D-dimer. J. Am. Coll. Cardiol..

[59] Yamada Y (2020). Preoperative plasma d-dimer level is a useful prognostic marker in ovarian cancer. J. Obstet. Gynaecol..

[60] Li L (2021). Utility of preoperative inflammatory markers to distinguish epithelial ovarian cancer from benign ovarian masses. J. Cancer..

[61] Giannakeas V, Narod SA (2021). Incidence of cancer among adults with thrombocytosis in Ontario, Canada. JAMA Netw. Open..

